# Different Methods of Minimally Invasive Esophagojejunostomy After Total Gastrectomy for Gastric Cancer: Outcomes from Two Experienced Centers

**DOI:** 10.1245/s10434-023-13771-2

**Published:** 2023-07-13

**Authors:** Yongjia Yan, Daohan Wang, Kelly Mahuron, Xi Wang, Li Lu, Zhicheng Zhao, Laleh Melstrom, Chuan Li, I. Benjamin Paz, Jian Liu, Yuman Fong, Weidong Li, Weihua Fu, Yanghee Woo

**Affiliations:** 1 Division of Surgical Oncology, Department of Surgery, City of Hope National Medical Center, Duarte, CA USA; 2 Department of General Surgery, Tianjin Medical University General Hospital, Tianjin, China; 3 Cancer Immunotherapeutics Program, Beckman Research Institute, City of Hope, Duarte, CA USA

**Keywords:** Esophagojejunostomy, Gastric cancer, Quality-of-life, Minimally invasive total gastrectomy, Esophagojejunal reconstruction, Intracorporeal esophagojejunostomy

## Abstract

**Background:**

Esophagojejunostomy after minimally invasive total gastrectomy (MITG) for gastric cancer (GC) is technically challenging. Failure of the esophagojejunal anastomosis can lead to significant morbidity, leading to short- and long-term quality of life (QoL) impairment or mortality. The optimal reconstruction method following MITG remains controversial. We evaluated outcomes of minimally invasive esophagojejunostomy after laparoscopic or robotic total gastrectomies.

**Methods:**

We retrospectively reviewed MITG patients between 2015 and 2020 at two high-volume centers in China and the United States. Eligible patients were divided into groups by different reconstruction methods. We compared clinicopathologic characteristics, postoperative outcomes, including complication rates, overall survival rate (OS), disease-free survival rate (DFS), and patient-reported QoL.

**Results:**

GC patients (*n* = 105) were divided into intracorporeal esophagojejunostomy (IEJ, *n* = 60) and extracorporeal esophagojejunostomy (EEJ, *n* = 45) groups. EEJ had higher incidence of wound infection (8.3% vs 13.3%, *P* = 0.044) and pneumonia (21.7% vs 40.0%, *P* = 0.042) than IEJ. The linear stapler (LS) group was inferior to the circular stapler (CS) group in reflux [50.0 (11.1–77.8) vs 44.4 (0.0–66.7), *P* = 0.041] and diarrhea [33.3 (0.0–66.7) vs 0.0 (0.0–66.7), *P* = 0.045] while LS was better than CS for dysphagia [22.2 (0.0–33.3) vs 11.1 (0.0–33.3), *P* = 0.049] and eating restrictions [33.3 (16.7–58.3) vs 41.7 (16.7–66.7), *P* = 0.029] at 1 year. OS and DFS did not differ significantly between LS and CS.

**Conclusions:**

IEJ anastomosis generated better results than EEJ. LS was associated with a better patient eating experience, but more diarrhea and reflux compared with CS. Clinical and patient-reported outcomes show the superiority of IEJ with the LS reconstruction method in MITG for GC.

**Supplementary Information:**

The online version contains supplementary material available at 10.1245/s10434-023-13771-2.

Although the overall diagnosis of gastric cancer (GC) is declining worldwide, the incidence and proportion of proximal GC are increasing. An estimated 20–40% of gastric tumors are now found in the upper portions of the stomach.^1,2^ For patients with proximal GC, a total gastrectomy (TG) with esophagojejunal (EJ) reconstruction is often required to achieve the oncological goal of R0 resection critical to every curative intent treatment for GC. However, the surgical techniques used to reconstitute the gastrointestinal tract with an EJ anastomosis can be technically challenging in a TG with EJ reconstruction, and can be associated with significant patient morbidity and mortality. The failure of EJ anastomoses leads to postoperative adverse events that have an immediate negative impact on the patient’s postoperative course with long-lasting quality-of-life impairments. In particular, the intrathoracic retraction of the distal esophagus, the narrow operative view through the esophageal hiatus, and the manipulation of the esophagus and small bowel are special considerations during minimally invasive approaches in order to create a tension-free and well-vascularized EJ anastomosis. While several methods for both laparoscopic and robotic esophagojejunostomy have been described, no consensus exists on the best reconstruction methods, and guidelines are absent.

Minimally invasive surgery (MIS) in the treatment of GC has improved postoperative outcomes, such as less pain, better cosmetic results, faster recovery, and shorter hospital stays than open surgery.^3^ However, both open and MIS approaches to total gastrectomy risk potentially severe complications associated with EJ anastomotic failure, such as leakage, pneumonia, pleural effusions, bleeding, stenosis, and death.^4,5^ Hence, in the beginning, MIS total gastrectomy often adopted an 8- to 10-cm midline incision to complete an extracorporeal esophageal-jejunal anastomosis using the same technique as conventional open surgery. For MIS radical total gastrectomy, the technical complexity of reconstruction remains a significant barrier to the wide adoption of both robotic and laparoscopic approaches. A better understanding of the comparative outcomes associated with the different minimally invasive methods for creating an EJ anastomosis after TG is needed to optimize surgical techniques and improve GC patient outcomes.

## Methods

### Patients

**Fig. 1:**
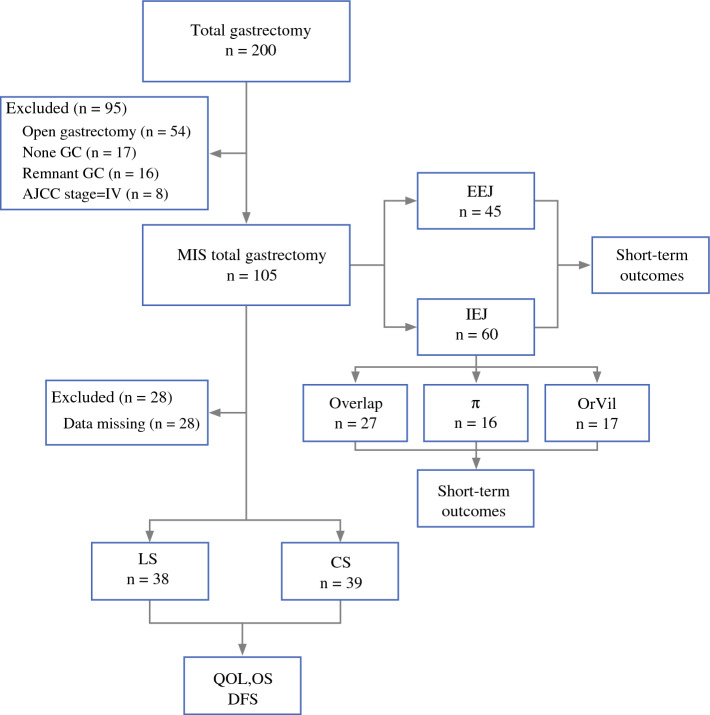
Flow chart demonstrating patient selection and grouping

Prospectively maintained data from all patients who underwent total gastrectomy at two high-volume centers in China and the United States, between June 2015 and June 2020 were retrospectively analyzed. Patients with diagnoses other than adenocarcinoma, and those with open gastrectomy, remnant, or stage IV GC were excluded. According to the anastomosis technique, patients were divided into subgroups: EEJ vs IEJ, overlap cohort vs π-shape cohort vs OrVil cohort, and LS vs CS (Fig. 1). All surgeries were performed by experienced surgeons, who determined the surgical approach (laparoscopic and robotic) by their preference after thorough discussion with the patients, who also consented to video recording of their operations. Videos of operative procedures were evaluated between the two corresponding authors for quality control. Data collection was approved by the Institutional Review Boards of the participating centers.

### Surgical Technique (see videos)

#### Functional End-to-End Anastomosis (π-Shaped)

After complete transhiatal mobilization of the distal esophagus (at least 6–8 cm), we looped the esophagogastric junction (EGJ) with a No. 8 urethral catheter and used it to retract the esophagus downward and into the abdomen. We created two enterotomies: one on the right side of the distal esophagus and the other on the anti-mesentery side of the jejunum 20–30 cm distal to the Treitz ligament. Under the guidance of the gastric tube, we inserted a 60-mm linear stapler into the esophagus and jejunum to create a functional end-to-end esophagojejunostomy and close the common opening with another 60-mm linear stapler.

#### Side-to-Side Anastomosis (Overlap)

After completion of the resection and specimen extraction, the pneumoperitoneum was re-established. We identified the ligament of Trietz (LOT) and divided the jejunum at least 20 cm distally. Then we opened the anti-mesentery side of the distal jejunum 5 cm from the distal jejunal staple line. Two barbed threads were sutured on both sides of the esophageal stump and used for traction to pull the distal esophagus in the abdomen with the help of the assistant. Then an entry hole was made in the center of the esophageal stump. A 45-mm linear stapler was placed into the jejunum and esophagus. We constructed an anteroposterior anastomosis of the esophagus and jejunum and closed the common enterotomy with sutures with reinforcement using pre-sutured barbed threads.

#### End-to-Side Anastomosis (EEA) (OrVil)

After dividing the distal esophagus using a linear endoGIA Tristapler (purple, loaded with 3.0-, 3.5-, and 4.0-mm staples), the total gastrectomy specimen was extracted through an extended assist port site, after which a frozen section was used to confirm histologically negative esophageal margins. Then the OrVil orogastric tube was inserted transorally until the tip of the tube reached the center of the distal esophageal staple line. It was passed through a small hole made with a sharp instrument anteriorly to the staple line. The tip of the tube was passed from robotic instruments to the bedside assistant and pulled gently into the abdomen and out of the abdomen through the assist port until the anvil was visible. The tube was disconnected from the anvil and removed. Then we extended the 15-mm assist port on the left side of the patient’s abdomen to slightly larger than 25 mm and intubated the shaft of the EEA. The EEA was mated to the anvil to create an end-to-side esophageal-jejunal anastomosis with the circular stapler. The open end of the jejunum was closed with a linear stapler.

### Data Collection

Clinicopathological characteristics, perioperative outcomes, and postoperative morbidity and mortality were previously obtained from the medical records. The quality of life (QoL) was assessed by the European Organization for Research and Treatment of Cancer QoL questionnaire (QLQ-C30, Chinese version) and GC module QoL questionnaire (QLQ-STO22, Chinese version) scales at pre-operation and at 1 year for circular stapler (CS) and linear stapler (LS) for patients.^6^ To reduce the bias caused by different lifestyles between Eastern and Western countries, we only included the QoL data of Chinese patients in the study. The 17 cases from the U.S. were excluded as data missing since they did not have the QoL questionnaire. The same population was followed up for overall survival rate (OS) and disease-free survival rate (DFS). Six people were lost at 1-year follow-up in the CS group, while five were lost in the LS group.

### Statistical Analysis

SPSS version 26 statistical software (Chicago, IL, USA) was used to perform the statistical analysis. Continuous variables are presented as mean ± SD and were compared among cohorts using one-way analysis of variance (ANOVA) or the Kruskal-Wallis H test. Categorical variables were analyzed by chi-square or Fisher’s exact test. *P* < 0.05 was considered statistically significant.

## Results

### Patient Clinicopathological Characteristics and Subgroup Analyses

A flow diagram demonstrating patient selection and grouping is shown in Fig. 1. Of the initially identified 200 patients who underwent either laparoscopic or robotic total gastrectomy, 95 patients were excluded for not having a GC diagnosis, or for having remnant cancer, stage IV disease, or for receiving an open operation. Based on these criteria, 105 patients were enrolled in this study. Table 1 shows the patient clinicopathological characteristics for the IEJ and EEJ groups. No significant differences related to sex, body mass index (BMI), American Society of Anesthesiologists (ASA) score, comorbidity, abdominal surgery history, long diameter of tumor, Lauren classification, or TNM stage were observed. Patients in the IEJ group were younger than those in the EEJ group (*P* = 0.030) and accepted neoadjuvant therapy before surgery more readily (21.7% vs 4.4%, *P* = 0.013).

**Table 1:** Clinicopathological characteristics between IEJ and EEG

Variable	IEJ (*n* = 60)	EEJ (*n* = 45)	*P*
Sex (male, *n,* %)	38 (63.3)	31 (68.9)	0.553
Age (year)	61.4±12.6	64.5±9.2	0.030
BMI (kg/m^2^)	25.6±4.1	22.8±3.4	0.764
ASA score (*n*, %)			0.060^*^
1	6 (10.0)	3 (6.7)	
2	15 (25.0)	22 (48.9)	
3	37 (61.7)	20 (44.4)	
4	2 (3.3)	0 (0.0)	
Comorbidity (*n*, %)			
Diabetes	10 (16.7)	12 (26.7)	0.213
CVD	20 (33.3)	18 (40.0)	0.482
Abdominal surgery history (*n*, %)	7 (11.7)	9 (20.0)	0.240
Neoadjuvant therapy (*n*, %)	13 (21.7)	2 (4.4)	0.013
Long diameter of tumor (cm)	4.5±2.6	5.8±2.3	0.455
Lauren classification (intestinal, *n*, %)	33 (55.0)	25 (55.6)	0.955
Depth of infiltration (*n*, %)			0.494
T1	7 (11.7)	3 (6.7)	
T2	12 (20.0)	11 (24.4)	
T3	10 (16.7)	4 (8.9)	
T4	31 (51.7)	27 (60.0)	
Lymph node status (*n*, %)			0.467
N0	22 (36.7)	13 (28.9)	
N1	15 (25.0)	8 (17.8)	
N2	9 (15.0)	8 (17.8)	
N3	14 (23.3)	16 (35.6)	
TNM (*n*, %)			0.242
I	12 (20.0)	4 (8.9)	
II	18 (30.0)	18 (40.0)	
III	30 (50.0)	23 (51.1)	

*Fisher test. *BMI*, body mass index, *ASA*, American Society of Anesthesiologists, *CVD*, cardio vascular disease.

Subsequently, according to the reconstruction methods, we divided the IEJ cohort into 3 subgroups (overlap, π-shaped, and OrVil anastomosis). We found a significant difference in age, neoadjuvant therapy, and TNM stage (*P* < 0.05). No significant differences were observed among the 3 groups in terms of sex, BMI, ASA score, comorbidity, and so on (*P* > 0.05) (Supplementary Table 1).

### Operation Details and Postoperative Morbidity and Mortality

The operative findings, summarized in Table 2, show no significant difference between IEJ and EEJ (*P* > 0.05). However, we identified significant differences in postoperative outcomes between the two groups (Table 2). The most common postoperative morbidity was pneumonia which was higher in EEJ than in IEJ (40.0% vs 21.7%, *P* = 0.042). IEJ was associated with fewer wound infections (8.3% vs 22.2%, *P* = 0.044). The incidence of other types of morbidity was comparable between the two groups (*P* > 0.05). Severe postoperative complications, grade ≥ III, based on the Clavien-Dindo classification system, did not significantly differ between the two groups (*P* = 0.737). Two patients died in each group during hospitalization, and there were no significant differences between the two groups in terms of mortality (*P* = 0.769) (Table 2).

**Table 2:** Operative findings and postoperative morbidity and mortality between IEJ and EEJ

Variable	IEJ (*n* = 60)	EEJ (*n* = 45)	*P*
Operation time (min, median)	320 (213–544)	300 (210–400)	0.100
Estimated blood loss (ml, median)	100 (5–500)	10 (10–200)	0.160
Number of dissected nodes (*n*, median)	39 (15–84)	45 (17–70)	0.261
Surgical morbidity (*n*, %)	22 (36.7)	25 (55.6)	0.054
Wound infection	5 (8.3)	10 (22.2)	0.044
Anastomotic stenosis	2 (3.3)	6 (13.3)	0.056
Abdominal bleeding	0 (0.0)	2 (4.4)	0.099^*^
Anastomotic leakage (≥ B grade)	6 (10.0)	2 (4.4)	0.288
Pancreatic fistula	4 (6.7)	8 (17.8)	0.077
Ileus	2 (3.3)	6 (13.3)	0.056
Abdominal infection	4 (6.7)	5 (11.1)	0.421
Pleural effusion	4(6.7)	2 (4.4)	0.627
Other (*n*, %)	24 (40.0)	29 (64.4)	0.013
Pneumonia	13 (21.7)	18 (40.0)	0.042
Urinary system	4 (6.7)	7 (15.6)	0.141
Deep vein thrombosis	8 (13.3)	10 (22.2)	0.232
Clavien-Dindo grade ≥ IIIa (*n*, %)	13 (21.7)	11 (24.4)	0.737
Mortality (*n*, %)	2 (3.3)	2 (4.4)	0.769

^*^Fisher test

Amongst the three major techniques used in IEJ, the median operation time in the OrVil group was the longest, although the difference did not reach statistical significance (*P* = 0.376). In addition, the estimated blood loss and the number of dissected nodes were similar among the three groups (*P* > 0.05) (Supplementary Table 2). Further analyses showed no significant differences in postoperative morbidity and mortality associated with the type of IEJ reconstruction (Supplementary Table 3).

### EORTC QLQ-C30 and QLQ-STO22

**Fig. 2:**
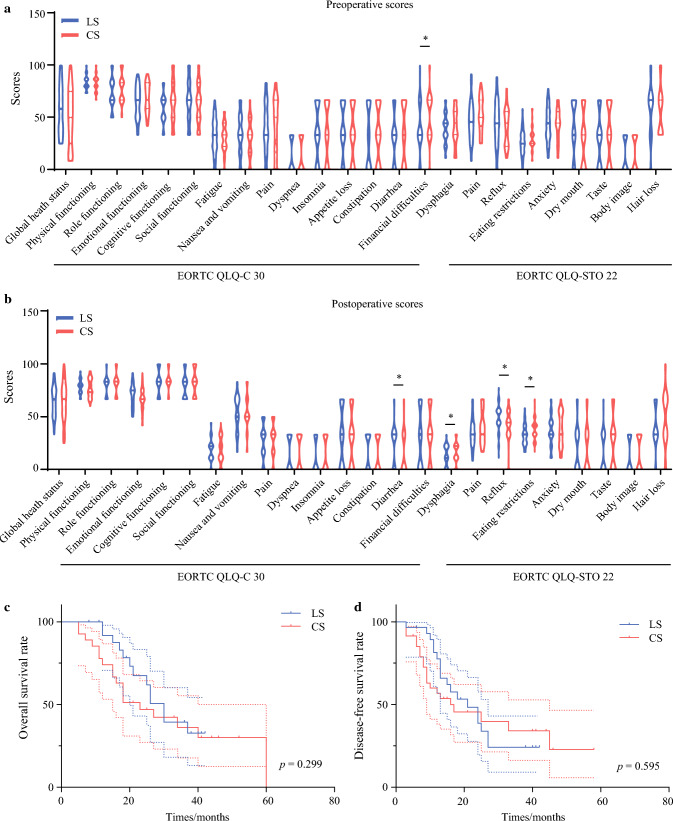
Quality of life changes and survival analysis for different EJ reconstruction methods. The change of EORTC QLQ-C30 and QLQ-STO22 score in LS and CS: **a** Preoperative QoL scores and **b** postoperative QoL scores at 1 year follow-up. **c**, **d** Survival curves by LS and CS: **c** overall survival and **d** disease-free survival. The *dashed lines* indicate the 95% confidence intervals

The chronological changes in scores of the EORTC QLQ-C30 and QLQ-STO22 symptom scales revealed no significant differences in any of the functioning parameters except financial difficulties before surgery [33.3 (0–100) vs 66.7 (0–100), *P* = 0.039] between the LS and CS groups (Fig. 2a). At the 1-year follow-up, patients in the LS group reported significantly more diarrhea [33.3 (0–66.7) vs 0 (0–66.7), *P* = 0.045] and higher reflux scores [50.0 (11.1–77.8) vs 44.4 (0–66.7), *P* = 0.041] than the CS group. The CS group reported poorer scores for dysphagia [22.2 (0–33.3) vs 11.1 (0–33.3), *P* = 0.049] and eating restrictions [41.7 (16.7–66.7) vs 33.3 (16.7–58.3), *P* = 0.029] (Fig. 2b). There were no significant differences in other reported QoL parameters between the two groups (Supplementary Table 4).

### Survival Analysis

The median follow-up time was 18 months (3 to 60 months). The Kaplan–Meier survival curves showed that the OS (Fig. 2c) and DFS (Fig. 2d) did not differ between the two groups. The 3-year OS was 43.5% and 44.9% for LS and CS, respectively (*P* = 0.299), and the 3-year DFS was 33.2% and 41.1% for LS and CS, respectively (*P* = 0.595).

## Discussion

With increasing incidences of upper and middle GC that require more TGs as the oncologically appropriate extent of stomach resection,^7,8^ and the positive results of CLASS02,^9^ JCOG1401,^10^ and KLASS03^11^ clinical trials that support the benefits of minimally invasive total gastrectomy (MITG), the surgeon’s ability to minimize perioperative complications and improve long-term patient outcome is imperative. The real-life experiences with MIS for proximal GC at our two high-volume specialized GC centers between 2015 and 2020 demonstrate that both laparoscopic and robotic TGs with various EJ techniques are safely and routinely performed. For the first time, we show the short-term benefits of IEJ with LS that reduced wound infections and pneumonia, and patient-reported advantages of less dysphagia and fewer eating restrictions compared with reduced incidence of reflux and diarrhea with the use of CS.

Studies evaluating methods of reconstructing the continuity of the esophago-intestinal tract after MITG have different outcomes, and the best technique for creating esophagojejunal anastomosis remains a topic of debate. A key challenge to interpreting the outcomes of these conflicting study results is in the details of various MITG with IEJ or EEJ procedures which may differ, such as length of the incision and operating exposure^12,13^ as well as surgeon-specific factors such as training, volume, and experience. Patients with obesity, smaller esophageal hiatus, or costal arch angle always significantly increase surgical difficulty and prolong the operative time because of the limited surgical field exposure. IEJ can provide a clear view with a small incision. However, obtaining good surgical exposure and safely performing EEJ using a normal assisted incision is usually challenging. It is often necessary to extend the incision, which may lead to wound-related complications. In our study, the incidence of wound-related complications in EEJ was significantly higher than in IEJ. In contrast to our study, WH Han et al. showed that their overall complication rate was higher in the IEJ group than in the EEG group. They considered this might be related to the deficiency of technical proficiency and experience.^14^ Moreover, the leakage rate of IEJ is higher than that of EEJ, although it was not statistically significant. This is most likely due to patients in the IEJ group having higher BMI and ASA, both of which are factors predictive of EJ leaks. Moreover, considering that circular staplers have two rows of staples, while linear staplers have three rows, in theory, circular staplers are more prone to anastomotic leakage. This issue could contribute to higher IEJ leakage rates than found with EEJ. A meta-analysis consisting of 8 studies with 1883 patients showed no significant differences in operating time, anastomotic time, the length of resection margin, postoperative recovery, anastomosis-related complication, and overall complication between the two groups. But IEJ is more minimally invasive, with less blood loss and more significant lymph node dissections.^15^ Our study found that IEJ had significantly reduced pneumonia rates, perhaps attributable to younger patients.

**Fig. 3:**
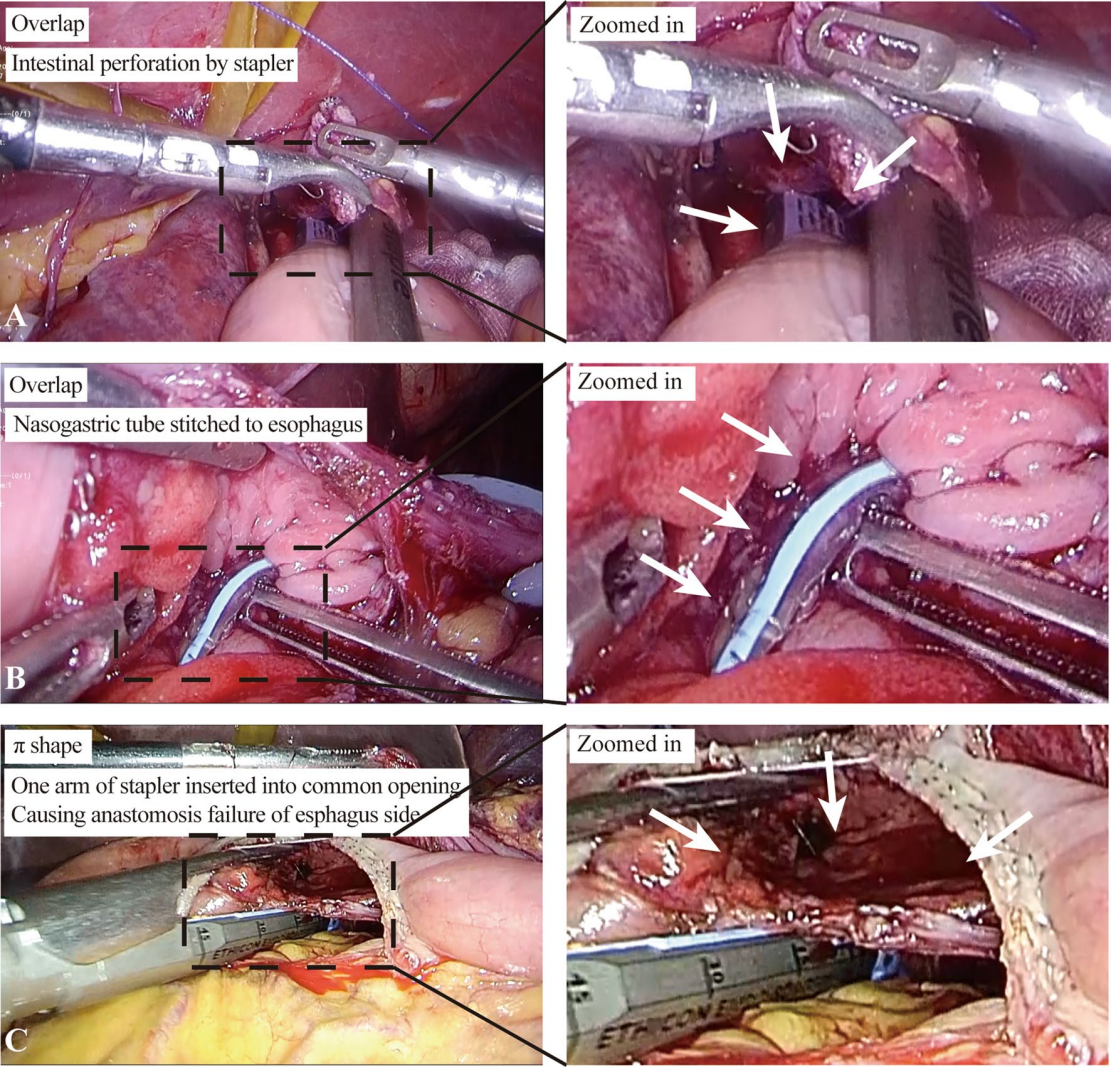
Esophagojejunal anastomosis. During overlap reconstruction, if the tension is high, the stapler will cause intestinal perforation (**a**). And before firing the stapler, the nasogastric tube for guidance should be unplugged, or it might get stitched (**b**). In the π-shaped anastomosis, if the common opening has not been closed, one arm of the stapler may slip into it and cause anastomosis failure (**c**). The white arrows are pointing at the intestinal perforation caused by the stapler (**a**), the stitched nasogastric tube (**b**), and the failure anastomosis due to the stapler entering the common opening (**c**), respectively. *Right side* shows magnified images from dashed-line boxes on *left side*

Overlap, π-shaped, and OrVil are commonly used in IEJ and have gradually been proposed and adopted by surgeons,^16–18^ but few studies have compared them. An LS is used in overlap and π-shaped techniques, which can be inserted into the abdominal cavity through a 12-mm trocar for anastomosis. The surgeon can obtain a good surgical field with the suspended liver and assistant retraction. However, the CS often needs an additional small incision or assist port extension, which is bulky and can block the operation field, resulting in increased difficulty and prolonged operation time. In this study, no significant difference was found in operation time, which may be attributed to the experienced surgical team. In addition, a successful anastomosis requires attention to many details; we have also experienced some cases of anastomosis failure, summarized in Fig. 3. The overlap and π-shaped anastomosis always need a longer esophagus and jejunum to safely complete side-to-side anastomosis. Completing a tension-free anastomosis with an R0 resection for patients with a higher tumor invasion is challenging. We preferred cutting the diaphragmatic foot and pulling down the esophagus to complete the anastomosis. In addition, two knotless barbs are sutured on the stapled line of the esophageal stump, which provides convenience for the assistant to pull the distal esophagus.^19^ Unlike the small intestine, the muscular and mucosal layers of the esophagus can be easily separated, so there is a risk of inserting one arm of the LS into a false lumen during EEJ, especially when the incision in the esophagus is small. In our study, when overlap and π-shaped anastomosis were performed, a nasogastric tube was inserted through the incision in the esophagus. Then the anvil of the LS was inserted into the esophagus, guided by the nasogastric tube. Therefore, we conducted a subgroup analysis to investigate differences between the different methods. There was no significant difference in postoperative complications between the three major techniques examined. Several previous studies differ from our findings. For example, Kawamura et al.^20^ found that overlap was related to fewer anastomotic complications than the OrVil procedure, especially regarding anastomotic stenosis. These findings are similar to those of Chen et al., who showed that overlap was superior to OrVil in the incidence of perioperative, postoperative, and anastomotic-related complications, the severity of complications, and the time of postoperative recovery.^21^ Furthermore, compared with overlap anastomosis, π-shaped anastomosis could reduce the operation and anastomosis time with the same results for postoperative complications, margin distance, etc.^22^ In our experience, minimally invasive IEJ techniques provide clear intra-abdominal and transhiatal views with a small incision. Thus, IEJ with linear staplers such as overlap and π-shaped anastomoses are preferred when possible. In terms of tumor location, tumors that do not involve the GEJ or cardia are the safest for these methods. Since overlap and π-shaped anastomoses always require exposure to a longer esophagus and jejunum to safely complete the side-to-side anastomosis, these IEJs are technically limited when more than 5 cm of the distal esophagus is resected due to tumors that involve the distal esophagus. The π-shaped anastomosis is generally not the first choice when the tumor location is too high to guarantee a negative margin, because one cannot get definitive proof of the negative margin before completing the esophagojejunostomy. π-shaped anastomosis is technically simpler than overlap in MIS surgery, but the margin cannot be checked before the anastomosis is finished. On the other hand, we can remove the specimen and ensure the margin is negative, then finish the overlap anastomosis. So, if the tumor is located in the esophagogastric junction and the upper edge is less than 2 cm from the cardia, we tend to perform an overlap anastomosis and check the margin during the operation; otherwise, we choose a π-shaped anastomosis. Moreover, the IEJ with OrVil can be helpful when the distal esophageal resection margin is high up in the mediastinum. The anvil can be placed using the NGT to guide and secure the anvil to the distal esophageal staple line. The mating of the shaft of the EEA and the anvil takes some experience. In case of stapler misfires or misaligned or mesenteric twisting of EJs, circular EJs are easier to salvage because a short segment of the distal esophagus requires resection. In failures of linear stapling of the EJ, a long segment of the distal esophagus can be damaged, and recovery or redo of the EJ becomes a significant challenge and may require a thoracic approach.

Linear and circular staplers are widely used in many medical centers during MITG. LS is associated with a low risk of anastomotic leakage, especially in MIS.^18,23,24^ However, most studies demonstrated that the safety and efficacy of the two staplers were similar during the operation. LS only had advantages in shortening the anastomotic time under MITG, and there was no significant difference in surgical outcomes or overall complications between the two groups.^20,25,26^ Compared with the 2-row CS, the 3-row LS enhanced anastomosis. The most significant difference between CS and LS is the inner diameter of the anastomosis. A 25-mm-diameter CS is the most frequently used in EEJ, and its diameter is not comparable to an LS with a 28- to 30-mm diameter. The different anastomotic diameters thus may affect the long-term outcomes. We explored the difference between LS and CS for long-term prognosis, including OS, DFS, and QoL. At first, we analyzed baseline data, and no significant differences between the two groups, except the TNM stage, were observed (Supplementary Table 5). Whether LS or CS, it did not affect the OS or DFS. We found some notable results for QoL. LS was associated with a better eating experience with a larger anastomosis but poor anti-reflux, and diarrhea at the 1-year follow-up. This may be attributed to drugs, emotions, gut microbiota, foods and diets, and abnormal intestinal activities after surgery.^27–29^ However, it is still unclear which factor mentioned above differs between the two groups and this topic needs further study.

The limitations of this study include the selection bias inherent in a retrospective study. Unfortunately, compared with IEJ, the number of patients receiving EEJ was lower and limited our ability to perform propensity score matching of baseline characteristics. Also, the pooling of data from the two institutions where all EEJs were performed by surgeons in China and all OrVil-assisted IEJs were performed in robotic gastrectomies in the United States complicates the interpretation of the study. Despite these limitations, the findings of our study demonstrate the outcome from real-life variations in surgical technique for EJ anastomoses after TG for GC amongst surgeons and institutions worldwide, and contribute to the knowledge about MITG with EJ anastomosis outcomes.

## Conclusions

Compared with EEJ, IEJ anastomosis is often associated with a low incidence of wound infection and pneumonia. All IEJ techniques are safe and similar in overall complications. LS may play an essential role in reducing dysphagia and eating restrictions, and CS may lead to a better score in diarrhea and reflux.

## Supplementary Information

Below is the link to the electronic supplementary material.

Supplementary file1 (DOCX 40 KB)

ESM2 (WMV 74302 kb)

ESM2 (WMV 76915 kb)
