# Supplementary material for: Different Methods of Minimally Invasive Esophagojejunostomy After Total Gastrectomy for Gastric Cancer: Outcomes from Two Experienced Centers
Source: Ann Surg Oncol. 2023 Jul 13;30(11):6718–27. doi: 10.1245/s10434-023-13771-2 (PMC10506935; doi:10.1245/s10434-023-13771-2)
Supplement: Supplementary file 1 — Supplementary file1 (DOCX 40 KB) [file 10434_2023_13771_MOESM1_ESM.docx]

**Supplemental Material**

eTable 1. Clinicopathological characteristics among overlap, π-shape and OrVil anastomosis

eTable 2. Operative findings among overlap, π-shape and OrVil anastomosis

eTable 3. Postoperative morbidity and mortality among overlap, π-shape and OrVil anastomosis

eTable 4. EORTC QLQ-C30 and QLQ-STO22

eTable 5. Clinicopathological characteristics between LS and CS

**eTable 1. Clinicopathological characteristics among overlap, π-shape and OrVil anastomosis**

| **Variable** | **Overlap**  **(n=27)** | **π-shape**  **(n=16)** | **OrVil**  **(n=17)** | ***P*** |
| --- | --- | --- | --- | --- |
| Sex (male, n, %) | 20(74.1) | 10(62.5) | 8(47.1) | 0.193 |
| Age (year) | 64.9±8.5 | 63.6±12.1 | 53.6±15.5^a^ | 0.009 |
| BMI (kg/m^2^) | 26.2±5.4 | 23.2±0.2 | 26.0±4.1 | 0.393 |
| ASA score (n, %) |  |  |  | 0.160* |
| 1 | 3(11.1) | 2(12.5) | 1(5.9) |  |
| 2 | 10(37.0) | 3(18.8) | 2(11.8) |  |
| 3 | 14(51.9) | 11(68.8) | 12(70.6) |  |
| 4 | 0(0) | 0(0) | 2(11.8) |  |
| Comorbidity (n, %) |  |  |  |  |
| Diabetes | 4(14.8) | 4(25.0) | 2(11.8) | 0.560 |
| CVD | 9(33.3) | 6(37.5) | 5(29.4) | 0.886 |
| Abdominal surgery history (n, %) | 4(14.8) | 1(6.3) | 2(11.8) | 0.699 |
| Neoadjuvant therapy (n, %) | 0(0) | 0(0) | 13(76.5) | <0.001* |
| Approach |  |  |  | 0.000* |
| Robotic | 10(37.0) | 0(0) | 15(88.2) |  |
| Laparoscopic | 17(63.0) | 16(100.0) | 2(11.8) |  |
| Long diameter of tumor (cm) | 5.2±2.7 | 3.6±2.2 | 4.3±2.6 | 0.149 |
| Lauren classification (intestinal, n, %) | 15(55.6) | 10(62.5) | 8(47.1) | 0.670 |
| Depth of infiltration (n, %) |  |  |  | 0.006 |
| T1 | 1(3.7) | 3(18.8) | 3(17.6) |  |
| T2 | 4(14.8) | 5(31.3) | 3(17.6) |  |
| T3 | 2(7.4) | 1(6.3) | 7(41.2) |  |
| T4 | 20(74.1) | 7(43.8) | 4(23.5) |  |
| Lymph node status (n, %) |  |  |  | 0.007* |
| N0 | 3(11.1) | 8(50.0) | 11(64.7) |  |
| N1 | 8(29.6) | 4(25.0) | 3(17.6) |  |
| N2 | 8(29.6) | 1(6.3) | 0(0.0) |  |
| N3 | 8(29.6) | 3(18.8) | 3(17.6) |  |
| TNM (n, %) |  |  |  | 0.004 |
| I | 2(7.4) | 5(31.3) | 5(29.4) |  |
| II | 4(14.8) | 6(37.5) | 8(47.1) |  |
| III | 21(77.8) | 5(31.3) | 4(23.5) |  |

* Fisher test. A. The difference was statistically significant between the OrVil and overlap (*P*=0.003), and π-shape (*P*=0.019)

BMI: Body Mass Index, ASA: American Society of Anesthesiologists, CVD: Cardio Vascular Disease

**eTable 2. Operative findings among overlap, π-shape and OrVil anastomosis**

| **Variable** | **Overlap**  **(n=27)** | **π-shape**  **(n=16)** | **OrVil**  **(n=17)** | ***P*** |
| --- | --- | --- | --- | --- |
| Operation time (min, median) | 320(220-390) | 310(230-410) | 358(213-544) | 0.376 |
| Estimated blood loss (ml, median) | 100(10-200) | 50(50-200) | 200(5-500) | 0.237 |
| Number of dissected nodes (n, median) | 40(25-84) | 38(27-64) | 42(15-66) | 0.725 |

**eTable 3. Postoperative morbidity and mortality among overlap, π-shape and OrVil anastomosis**

| **Variable** | **Overlap**  **(n=27)** | **π-shape**  **(n=16)** | **OrVil**  **(n=17)** | ***P*** |
| --- | --- | --- | --- | --- |
| Surgical morbidity (n, %) | 6(22.2) | 2(12.5) | 7(41.2) | 0.148 |
| Wound infection | 1(3.7) | 0(0.0) | 0(0.0) | 0.537* |
| Anastomotic stenosis | 0(0.0) | 0(0.0) | 1(5.9) | 0.276* |
| Abdominal bleeding | 0(0.0) | 0(0.0) | 0(0.0) | - |
| Anastomotic leakage | 1(3.7) | 0(0.0) | 3(17.6) | 0.090* |
| Pancreatic fistula | 2(7.4) | 1(6.3) | 0(0.0) | 0.528* |
| Ileus | 0(0.0) | 1(6.3) | 1(5.9) | 0.428* |
| Abdominal infection | 1(3.7) | 0(0.0) | 2(11.8) | 0.276* |
| Pleural effusion | 2(7.4) | 0(0.0) | 1(5.9) | 0.549* |
| Other (n, %) | 10(37.0) | 6(37.5) | 6(35.3) | 0.990 |
| Pneumonia | 4(14.8) | 2(12.5) | 0(0.0) | 0.260* |
| Urinary system | 1(3.7) | 2(12.5) | 1(5.9) | 0.529 |
| Deep vein thrombosis | 5(18.5) | 3(18.8) | 0(0.0) | 0.161* |
| Clavien-Dindo grade ≥IIIa (n, %) | 3(11.1) | 4(25.0) | 6(35.3) | 0.154 |
| Mortality (n, %) | 1(3.7) | 1(6.3) | 0(0.0) | 0.600* |

* Fisher test

**eTable 4. EORTC QLQ-C30 and QLQ-STO22**

| **Variable** | **Preoperative scores** | | | **Postoperative scores** | | |
| --- | --- | --- | --- | --- | --- | --- |
|  | **LS**  **(n=38)** | **CS**  **(n=39)** | ***P*** | **LS**  **(n=38)** | **CS**  **(n=39)** | ***P*** |
| QLQ-C30 (median) |  |  |  |  |  |  |
| Global health status | 58.3(25.0-100.0) | 50.0(8.3-100.0) | 0.268 | 66.7(33.3-91.7) | 66.7(25.0-100.0) | 0.849 |
| Functioning scales |  |  |  |  |  |  |
| Physical functioning | 80.0(73.3-100.0) | 86.7(66.7-100.0) | 0.792 | 80.0(66.7-93.3) | 73.3(60.0-93.3) | 0.399 |
| Role functioning | 66.7(50.0-100.0) | 83.3(50.0-100.0) | 0.256 | 83.3(66.7-100.0) | 83.3(66.7-100.0) | 0.481 |
| Emotional functioning | 66.7(33.3-91.7) | 66.7(41.7-91.7) | 0.265 | 75.0(50.0-91.7) | 66.7(41.7-91.7) | 0.106 |
| Cognitive functioning | 66.7(33.3-83.3) | 66.7(33.3-100.0) | 0.150 | 83.3(66.7-100.0) | 83.3(66.7-100.0) | 0.635 |
| Social functioning | 66.7(33.3-100.0) | 66.7(33.3-100.0) | 0.543 | 83.3(66.7-100.0) | 83.3(66.7-100.0) | 0.293 |
| Symptoms scales |  |  |  |  |  |  |
| Fatigue | 33.3(0.0-66.7) | 33.3(0.0-55.6) | 0.843 | 22.2(0.0-44.4) | 22.2(0.0-44.4) | 0.843 |
| Nausea and vomiting | 33.3(0.0-66.7) | 33.3(0.0-66.7) | 0.425 | 50.0(16.7-83.3) | 50.0(16.7-83.3) | 0.738 |
| Pain | 33.3(0.0-83.3) | 50.0(0.0-83.3) | 0.659 | 33.3(0.0-50.0) | 33.3(0.0-50.0) | 0.422 |
| Dyspnea | 0.0(0.0-33.3) | 0.0(0.0-33.3) | 0.896 | 0.0(0.0-33.3) | 33.3(0.0-33.3) | 0.572 |
| Insomnia | 33.3(0.0-66.7) | 33.3(0.0-66.7) | 0.770 | 0.0(0.0-33.3) | 0.0(0.0-33.3) | 0.556 |
| Appetite loss | 33.3(0.0-66.7) | 33.3(0.0-66.7) | 0.471 | 33.3(0.0-66.7) | 33.3(0.0-66.7) | 0.927 |
| Constipation | 33.3(0.0-66.7) | 33.3(0.0-66.7) | 0.672 | 33.3(0.0-33.3) | 0.0(0.0-33.3) | 0.423 |
| Diarrhea | 33.3(0.0-66.7) | 33.3(0.0-66.7) | 0.574 | 33.3(0.0-66.7) | 0.0(0.0-66.7) | 0.045 |
| Financial difficulties | 33.3(0.0-100.0) | 66.7(0.0-100.0) | 0.011 | 33.3(0.0-66.7) | 33.3(0.0-66.7) | 0.363 |
| QLQ-STO22 (median) |  |  |  |  |  |  |
| Dysphagia | 44.4 (11.1-66.7) | 44.4(11.1-66.7) | 0.693 | 11.1(0.0-33.3) | 22.2(0.0-33.3) | 0.049 |
| Pain | 45.8(8.3-91.7) | 50.0(25.0-83.3) | 0.088 | 33.3(8.3-66.7) | 33.3(16.7-66.7) | 0.325 |
| Reflux | 44.4(0.0-88.9) | 44.4(11.1-77.8) | 0.333 | 50.0(11.1-77.8) | 44.4(0.0-66.7) | 0.041 |
| Eating restrictions | 25.0(0.0-58.3) | 25.0(8.3-58.3) | 0.254 | 33.3(16.7-58.3) | 41.7(16.7-66.7) | 0.029 |
| Anxiety | 44.4(11.1-77.8) | 44.4(11.1-66.7) | 0.662 | 33.3(11.1-66.7) | 33.3(11.1-66.7) | 0.815 |
| Dry mouth | 33.3(0.0-66.7) | 33.3(0.0-66.7) | 0.096 | 16.7(0.0-66.7) | 0.0(0.0-66.7) | 1.000 |
| Taste | 33.3(0.0-66.7) | 33.3(0.0-66.7) | 0.405 | 0.0(0.0-66.7) | 33.3(0.0-66.7) | 0.083 |
| Body image | 0.0(0.0-33.3) | 33.3(0.0-33.3) | 0.568 | 0.0(0.0-33.3) | 0.0(0.0-33.3) | 0.563 |
| Hair loss | 66.7(0.0-100.0) | 66.7(33.3-100.0) | 0.605 | 33.3(0.0-66.7) | 50.0(0.0-100.0) | 0.251 |

**eTable 5. Clinicopathological characteristics between LS and CS**

| **Variable** | **LS**  **(n=38)** | **CS**  **(n=39)** | ***P*** |
| --- | --- | --- | --- |
| Sex (male, n, %) | 28(73.7) | 26(66.7) | 0.501 |
| Age (year) | 64.2±10.3 | 64.2±9.7 | 0.629 |
| BMI (kg/m^2^) | 25.7±5.1 | 22.4±3.2 | 0.407 |
| ASA score (n, %) |  |  | 0.278^#^ |
| 1 | 4(10.5) | 2(5.1) |  |
| 2 | 13(34.2) | 20(51.3) |  |
| 3 | 21(55.3) | 17(43.6) |  |
| 4 | 0(0.0) | 0(0.0) |  |
| Comorbidity (n, %) |  |  |  |
| Diabetes | 7(18.4) | 11(28.2) | 0.310 |
| CVD | 14(36.8) | 14(35.9) | 0.931 |
| Abdominal surgery history (n, %) | 5(13.2) | 7(17.9) | 0.562 |
| Neoadjuvant therapy (n, %) | 0(0.0) | 2(5.1) | 0.157^#^ |
| Long diameter of tumor (cm) | 4.7±2.7 | 5.8±2.4 | 0.673 |
| Lauren classification (intestinal, n, %) | 20(52.6) | 23(59.0) | 0.575 |
| Depth of infiltration (n, %) |  |  | 0.668^#^ |
| T1 | 4(10.5) | 2(5.1) |  |
| T2 | 7(18.4) | 11(28.2) |  |
| T3 | 3(7.9) | 3(7.7) |  |
| T4 | 24(63.2) | 23(59.0) |  |
| Lymph node status (n, %) |  |  | 0.752 |
| N0 | 9(23.7) | 11(28.2) |  |
| N1 | 9(23.7) | 6(15.4) |  |
| N2 | 9(23.7) | 8(20.5) |  |
| N3 | 11(28.9) | 14(35.9) |  |
| TNM (n, %) |  |  | 0.035 |
| I | 7(18.4) | 3(7.7) |  |
| II | 6(15.4) | 16(41.0) |  |
| III | 25(65.8) | 20(51.3) |  |

* Fisher test. BMI: Body Mass Index, ASA: American Society of Anesthesiologists, CVD: Cardio Vascular Disease
